# Effects of Monensin and Rapamycin Combination Therapy on Tumor Growth and Apoptosis in a Xenograft Mouse Model of Neuroblastoma

**DOI:** 10.3390/antibiotics12060995

**Published:** 2023-06-01

**Authors:** Sema Serter Kocoglu, Fatma Bahar Sunay, Pakize Nur Akkaya

**Affiliations:** Department of Histology and Embryology, Faculty of Medicine, Balikesir University, 10145 Balikesir, Türkiye; sunay@balikesir.edu.tr (F.B.S.); nur.akkaya@balikesir.edu.tr (P.N.A.)

**Keywords:** monensin, rapamycin, combination therapy, neuroblastoma, mTOR pathway, apoptosis, in vivo, *Streptomyces cinnamonensis*

## Abstract

Neuroblastoma is the most common pediatric solid tumor originating from the neural crest. New treatment options are needed to improve treatment outcomes and the survival of patients with neuroblastoma. Monensin is an ionophore antibiotic with antiparasitic, antibacterial, and anticancer properties isolated from *Streptomyces cinnamonensis*. The aim of this study was to investigate the therapeutic effects of single and combined monensin and rapamycin treatments on mTOR (mammalian target of rapamycin) signaling pathway-mediated apoptosis and tumor growth in an SH-SY5Y neuroblastoma cell xenograft model. Control, monensin, rapamycin, and monensin + rapamycin groups were formed in the xenograft neuroblastoma model obtained from CD1 nude mice, and tumor volumes and animal weights were recorded throughout the treatment. In xenograft neuroblastoma tumor tissues, apoptosis was determined by TUNEL (terminal deoxynucleotidyl transferase-mediated dUTP nick end-labeling) and cleaved-caspase 3 immunohistochemistry, and PI3K (phosphoinositide-3-kinase)/AKT/mTOR expression was determined by the immunohistochemistry and immunofluorescence methods. The combination of monensin and rapamycin was to reduce the growth of xenograft neuroblastoma tumor tissues, trigger apoptosis, and suppress the expression of PI3K/AKT/mTOR. A significant increase in apoptotic cell rate was demonstrated in the combination group, supported by cleaved-caspase 3 immunohistochemistry results. In addition, it was reported that the combination treatment regime triggered apoptosis by reducing the expression of phosphorylated PI3K/AKT/mTOR. Our preclinical results may be a precursor to develop new therapeutic approaches to treat neuroblastoma.

## 1. Introduction

Neuroblastoma is the most common extracranial solid tumor in infants and children, comprising 8% to 9% of all childhood tumors. Neuroblastomas originate from the neural crest. Under normal conditions, neural crest cells originate from the neuroectoderm, then migrate to the dorsolateral side of the neural tube and differentiate into the cells, tissues, and organs of the sympathetic nervous system. However, in some cases, some defects that occur during the migration, differentiation, and maturation of neural crest cells can lead to the formation of neuroblastoma [1]. Most primary tumors are located in the adrenal gland [2]. Neuroblastomas comprise 15% of childhood cancer-related deaths, and the mean age at diagnosis is 22 months [3]. According to reports, 40% of newly diagnosed patients present at high risk, and most of the disease enters the metastasis stage [4]. The treatment options currently used for neuroblastoma are surgery, chemotherapy, radiotherapy, immunotherapy, and targeted therapy [5,6]. The prognosis of the disease varies with age, tumor grade, and various biological features, as well as amplification of the MYCN (MYCN Proto-Oncogene, BHLH Transcription Factor) oncogene [1]. Neuroblastoma is a biologically and clinically heterogeneous tumor, and the disease stage varies according to tumor characteristics. In addition, patients suffer from long-term treatment-related side effects [2]. Therefore, clinically relevant in vivo models are needed to investigate neuroblastoma disease’s underlying mechanisms and develop new therapeutic applications.

Monensin is an ionophore antibiotic with antiparasitic, antibacterial, and anticancer properties isolated from Streptomyces cinnamonensis [7]. Its chemical formula is C_36_H_61_NaO_11_ and its molecular weight is 692.86 g/mol (Abcam, cat. no: ab120499, Boston, MA, USA). It has antimicrobial properties because it can form pseudomacrocyclic complexes with metal cations and transport them across cellular membranes, disrupting the NA^+/^K^+^ balance and leading them to cell death. It is widely preferred to be used in the nutrition of ruminant animals [8]. Recent studies have shown that monensin inhibits proliferation and triggers apoptosis in pancreatic, breast, ovarian, and other cancer cells [9,10,11,12]. However, monensin’s effects and target mechanisms in an in vivo neuroblastoma cancer model have not been demonstrated before.

The mTOR signaling pathway is an essential regulator of cell development and metabolism. Rapamycin is a metabolite with antifungal, antiparasitic, and antiproliferative properties isolated from Streptomyces hygroscopicus, and is a specific mTOR inhibitor [13]. Its molecular formula is C_51_H_79_NO_13_, and its molecular weight is 914.17 g/mol (MedChemEkspress, cat. no: HY-10219, Newark, NJ, USA). It is an allosteric inhibitor of mTOR, a protein kinase known to be dysregulated in cancer and metabolic disorders. It was approved by the FDA in 1999 as an immunosuppressant [14]. The in vitro antitumor effects of rapamycin have been reported in various cancer lines and have been shown to inhibit cell proliferation in 80% of breast cancer cell lines [15]. Rapamycin has been shown to inhibit the proliferation of pancreatic cancer cells [16]. Rapamycin and its rapalogs have also been used in many clinical trials. Phase 1 studies have shown that mTOR inhibitors have no immunosuppressive effects and can tolerate drug-related toxic effects [17]. The overall response rate to treatment with an mTOR inhibitor (CCI-779) was 38% in patients with recurrent mantle cell lymphoma, one of the promising phase 2 studies [18]. Rapamycin, in combination with paclitaxel, has been shown to reduce tumor volume in a breast cancer xenograft model [19]. In another study, Marimpietri et al. reported the therapeutic effects of rapamycin in combination with vinblastine on human neuroblastoma development, apoptosis, and angiogenesis [20]. While the effects of rapamycin-based therapy are modest, combination therapies are promising due to their potential to increase drug efficacy by inhibiting multiple targets and delaying the emergence of drug resistance [13].

The aim of this study was to investigate the therapeutic effects of single and combination treatments of monensin and rapamycin on PI3K/AKT/mTOR signaling pathway-mediated apoptosis and tumor growth in the SH-SY5Y human neuroblastoma cell xenograft model.

## 2. Results

### 2.1. Combination Treatment of Monensin and Rapamycin Reduces Xenograft Neuroblastoma Tumor Development

**Figure 1:**
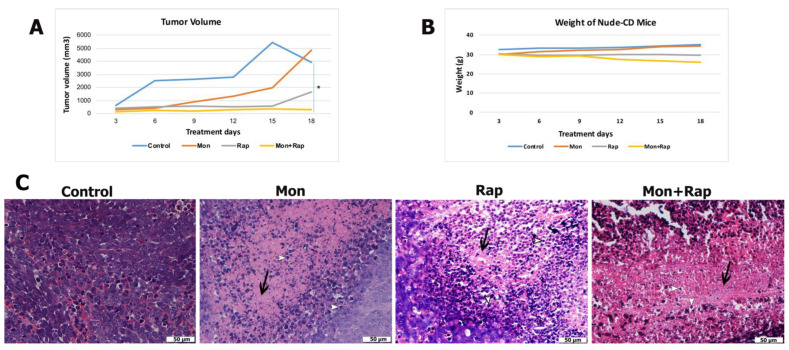
In vivo effects of single and combined administrations of monensin (mon) and rapamycin (rap) in the SH-SY5Y mouse xenograft model. (**A**) Tumor volume and (**B**) body weight changes of mice were measured before treatment. Data are presented as mean ± SD (*n* = 4). * indicates statistical differences of *p* < 0.05, respectively, compared to control group cells. (**C**) Histopathological changes in tumor tissue were evaluated by hematoxylin–eosin staining. Original magnification: 40× scale bar = 50 µm). Intense fibrous tissue formation between cells and increased apoptosis was observed in the single and combination treatment groups. Fibrous tissue is stained pink with eosin (black arrow), whereas hematoxylin stains chromatin in apoptotic cells and is evaluated according to cell morphology (white arrowhead). Intense changes, cell shrinkage, condensation of chromatin, shrinkage of the nucleus, and division into parts (white arrowhead).

The in vivo effects of monensin, rapamycin, and combination groups were evaluated in the xenograft tumor model created using CD1 nude (immundefficient) mice. There was no significant difference between the body weights of mice in the control and treatment groups (Figure 1B).

When the tumor volumes of the groups were compared, it was determined that tumor growth decreased in the monensin, rapamycin, and monensin + rapamycin groups after 18 days of treatment compared to the control group (Figure 1A and Supplementary Figure S1). Moreover, tumor volume reduction in the combination group was significant compared to the control group (*p* = 0.03) (Figure 1A). With the results of this study, it was shown for the first time that the combined use of monensin and rapamycin in the xenograft neuroblastoma tumor model created in mice caused a significant reduction in tumor size without causing significant toxicity.

Tumor formation was confirmed in all experimental groups with hematoxylin and eosin-stained sections, and tumor morphology of different experimental groups was evaluated. In the control group, which did not receive any treatment, tumor cells were found to be quite dense, and their borders were unclear. It was observed that the number of tumor cells decreased, cell death increased, and fibrosis tissue intensified in the groups treated with monensin alone or rapamycin alone. In the combined treatment group, the number of tumor cells was lower, and the fibrous tissue was more dense compared to the other groups. Changes in tumor morphology supported that the treatment groups induced cell death (Figure 1C).

### 2.2. Combination Treatment of Monensin and Rapamycin Triggers Apoptosis in Xenograft Neuroblastoma Tumor Tissues

**Figure 2:**
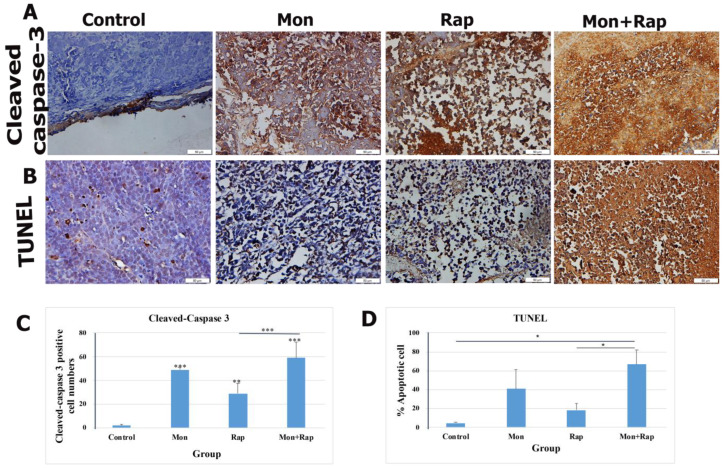
Evaluation of the effects of single and combined administrations of mon and rap on apoptosis in SH-SY5Y mouse xenograft model (**A**) by cleaved-caspase 3 immunohistochemistry (**B**) and TUNEL. (**C**) The number of cleaved-caspase 3 positive cells and (**D**) the percentage of apoptotic cells are indicated. Data are presented as mean ± SD (*n* = 4). Four different sections were analyzed for each animal. *, **, and *** indicate statistical differences of *p* < 0.05, *p* < 0.01, and *p* < 0.001, respectively, compared to control group cells. Original magnification: 40× (scale bar = 50 µm).

Apoptosis in xenograft tumor tissues was evaluated by TUNEL and cleaved-caspase 3 immunohistochemistry. A significant increase in apoptotic cell rate was observed in the monensin + rapamycin group compared to the control group (Figure 2B). While the rate of apoptotic cells was 4.5 ± 1.22% in the control group, these values were 40.89 ± 20.10%, 18.14 ± 7.2%, and 67.00 ± 15.18% (*p* = 0.022) in the monensin, rapamycin, and monensin + rapamycin groups, respectively (Figure 2D). In addition, the rate of apoptotic cells in the monensin + rapamycin group was significantly higher than in the rapamycin-administered group (*p* = 0.021) (Figure 2D). Cleaved-caspase 3 was found to be intensely expressed in the nuclei of tumor cells (Figure 2A). The number of cleaved-caspase 3 positive cells in tumor tissues was significantly increased in monensin, rapamycin, and monensin + rapamycin groups compared to the control group (Figure 2A). While the rate of cleaved-caspase 3 positive cells was 2 ± 1.15% in the control group, it was 49 ± 2.21% in the monensin group and 29 ± 8.61% in the rapamycin group, and 59 ± 13.47% in the combination group (Figure 2C).

### 2.3. The Combination of Monensin and Rapamycin Reduces PI3K/AKT/mTOR Expression in Xenograft Neuroblastoma Tumor Tissues

**Figure 3:**
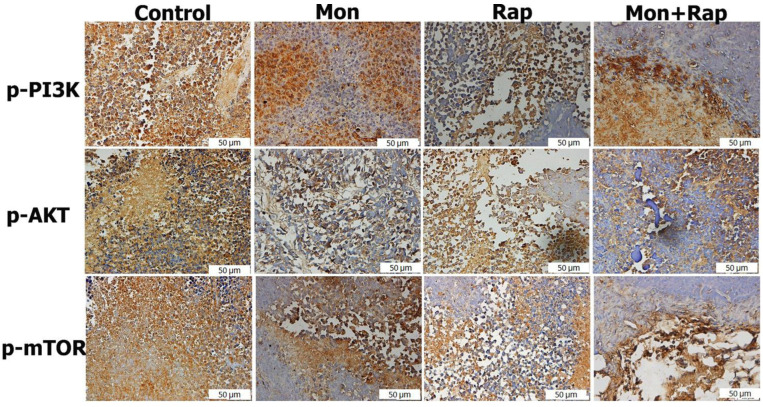
Immunohistochemistry evaluation of the effects of single and combined administrations of mon and rap on mTOR/PI3K/AKT signaling pathway in SH-SY5Y xenograft model. Original magnification: 40× (scale bar = 50 µm).

The expression of p-P3K, p-AKT, and p-mTOR in tumor tissues from xenograft mice was determined in the cytoplasm and nucleus of tumor cells. It was observed that the expression intensities of p-PI3K, p-AKT, and p-mTOR were decreased in the monensin, rapamycin, and combination groups compared to the control group (Figure 3).

**Figure 4:**
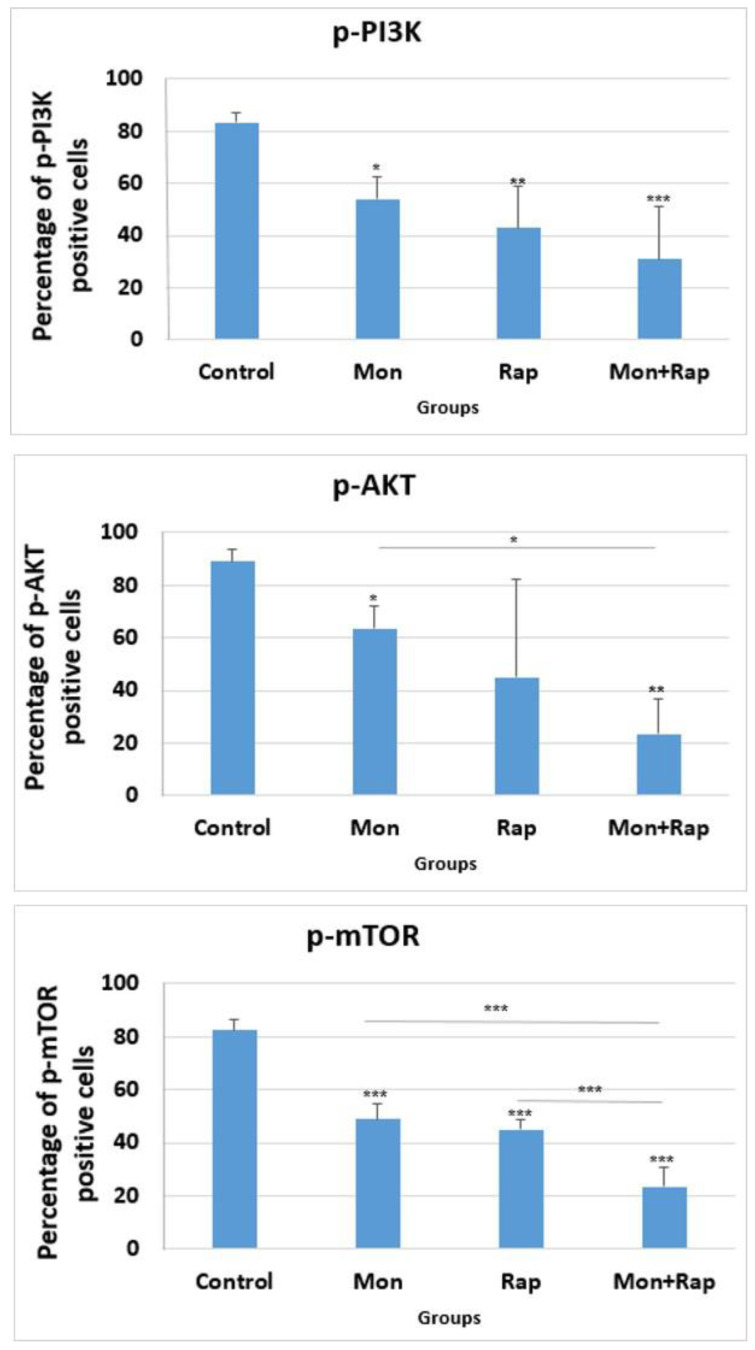
Statistical evaluation of the effects of single and combination groups on mTOR/PI3K/AKT signaling pathway in tumor tissues obtained from xenograft mice. Micrographs were taken using a light microscope at 40× magnification. All micrographs were analyzed with Image-J 2x.Ink software by scanning four nonoverlapping fields in each tissue and expressing the positive areas as a percentage of the total area. The percentage of cells expressing p-AKT, p-PI3K, and p-mTOR in each tissue was calculated with the Image-J software program. Data are presented as mean ± SD (*n* = 4). *, ** and *** indicate statistical differences of *p* < 0.05, *p* < 0.01 and *p* < 0.001, respectively, compared to control group cells.

The percentage of p-PI3K positive cells in tumor tissues was 83.5 ± 3.69%, 54 ± 8.75% (*p* = 0.042), 43 ± 15.85% (*p* = 0.006), and 31 ± 19.88% (*p* < 0.001) in the control, monensin rapamycin, and combination groups, respectively (Figure 4), while the number of cells expressing p-AKT in tumor tissues was 89 ± 4.7 in the control group, 63.5 ± 8.6% (*p* = 0.026), 45 ± 37.4% (*p* = 0.46), and 23.37% ± 13.21% (*p* = 0.006) in the monensin, rapamycin, and combination groups, respectively (Figure 4). The percentage of p-mTOR positive cells was 49 ± 5.83% (*p* < 0.001), 45 ± 3.46% (*p* < 0.001), and 23.5 ± 7.32 (*p* < 0.001) in the monensin, rapamycin and combination groups, respectively, while it was 82.5 ± 3.86% in the control group. The combination of monensin and rapamycin in tumor tissues significantly decreased p-PI3K, p-AKT, and p-mTOR expressions compared to the control group. In addition, p-mTOR expression was significantly decreased in the combination group compared to the monensin-alone and rapamycin-alone groups (*p* < 0.001). p-AKT expression was significantly decreased in the combination group compared to the monensin-alone group (*p* = 0.02) (Figure 4).

**Figure 5:**
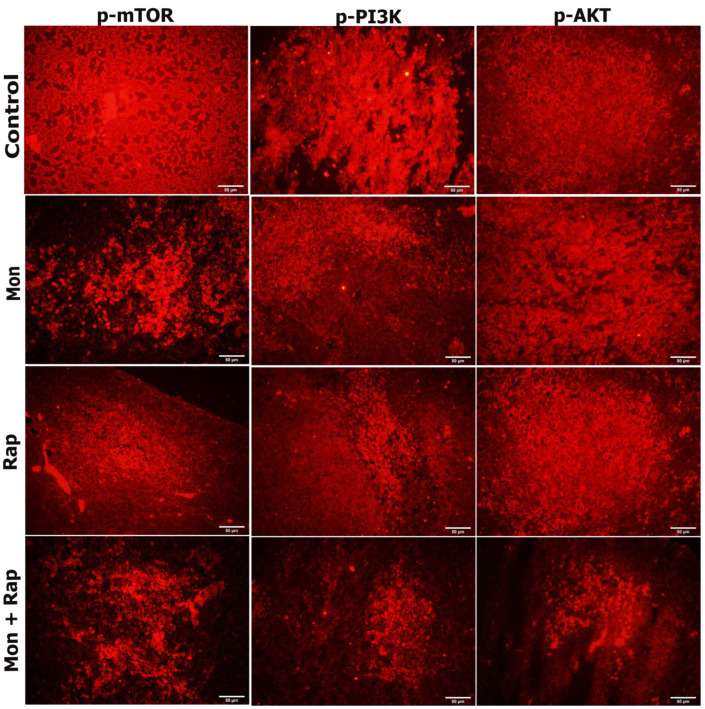
Evaluation of the effects of single and combined administrations of mon and rap on mTOR/PI3K/AKT signaling pathway in SH-SY5Y mouse xenograft model by immunofluorescence staining. Original magnification 40× (Scale bar = 50 µm).

The expression of p-mTOR, p-PI3K, and p-AKT was observed in SH-SY5Y cells of all experimental groups (Figure 5). The expression of p-mTOR, p-PI3K, and p-AKT was concentrated in the cytoplasm, membrane, and cytoplasmic extensions of the cell. While the staining intensity of p-mTOR, p-PI3K, and p-AKT expression was quite strong in the control group, a decrease was observed in the staining intensity when monensin + rapamycin were applied together (Figure 5). However, the change in staining intensity was not statistically evaluated.

## 3. Discussion

One of main goals of cancer research is to develop effective therapeutic approaches with limited side effects. Despite aggressive treatment options in treating neuroblastoma, the results are weak, and new therapeutic approaches are needed. There are two reasons why rapamycin was preferred for combination therapy with monensin in this study. (1) Rapamycin has been reported to sensitize some tumor cells to chemotherapeutics, and (2) it can be easily transferred to clinical therapy, as it is used clinically [20]. In this study, we report for the first time that monensin and rapamycin, when combined, exert a potential antitumor effect in a preclinical-associated in vivo xenograft neuroblastoma mouse model.

Johnsen et al. investigated the therapeutic efficacy of rapamycin on neuroblastoma development in vivo and showed that tumor volume was significantly reduced and caspase 3 activation was increased in the rapamycin group compared to the control group [21]. It has been reported that monensin significantly inhibits tumor growth at 8 and 16 mg/kg doses in an ovarian cancer xenograft model [11]. Another study reported that monensin (10 mg/kg) reduced tumor volume in the APC^+/Min^ intestinal cancer mouse model [22]. In our study, we examined for the first time the in vivo antitumor effects of both monensin and its combination with rapamycin in a xenograft SH-SY5Y model obtained from CD1 nude mice. We found that both the single therapy and combination groups reduced tumor volume. We also showed that combining monensin and rapamycin significantly reduced tumor volume compared to the control group. We evaluated the histopathological changes in the tumor tissue with hematoxylin–eosin staining. We showed that the number of tumor cells decreased and fibrous connective tissue areas increased in the monensin-alone, rapamycin-alone, and combination groups compared to the control group. Our study reports for the first time that combining monensin and rapamycin reduces tumor growth and induces cell death without significant toxicity.

Avoidance of apoptosis, the intrinsic cell death program, is a feature of human cancers, including neuroblastoma. In addition, the cytotoxic activity of anticancer treatments commonly used in the clinic, e.g., chemotherapy, gamma radiation, or immunotherapy, is mediated predominantly by triggering apoptosis in tumor cells. Therefore, a better understanding of the signaling pathways and molecules that govern apoptosis in neuroblastoma cells is expected to open new avenues for designing molecularly targeted therapies for neuroblastoma [23]. Gu et al. showed in vitro that monensin triggers apoptosis by reducing UBA2 expression in breast cancer cells and decreases cell proliferation and migration [10]. Kim et al. reported that monensin triggers mitochondrial ROS production and calcium-dependent apoptosis [12]. In our previous study, we reported that in vitro, monensin regulates PI3K/AKT signaling pathway-mediated cell proliferation and acts synergistically with rapamycin [24]. We focused our study on the hypothesis of the effects of the combination of monensin and rapamycin on apoptosis of xenograft neuroblastoma tumor cells in vivo and the role of the PI3K/AKT/mTOR pathway on this development in an advanced preclinical stage. In our study, the effects of monensin or rapamycin alone and their combinations on apoptosis were investigated for the first time by both TUNEL and cleaved-caspase 3 immunohistochemistry in a xenograft mouse model. The combination of monensin and rapamycin significantly increased the proportion of apoptotic cells and the number of cleaved-caspase 3 positive cells. In our study, it was shown for the first time that the combination of monensin and rapamycin significantly triggered apoptosis in an in vivo xenograft model and was confirmed by cleaved-caspase 3 expression.

Cell death plays an important role in every aspect of life. It plays a vital role in tissue homeostasis and the development of multicellular organisms. Various death mechanisms have been described in the last decade. The three that are best described are apoptosis, necroptosis, and pyroptosis. In addition to these, some of the other cell death pathways identified are as follows: autophagy-dependent cell death (ADCD), mitochondrial permeability transition pore (MPTP)-mediated necrosis, parthanatos, NETosis, and ferroptosis [25]. According to our results, rapamycin reduces tumor volume more than monensin, but monensin can trigger apoptosis more than rapamycin. In our study, we determined apoptosis, which is one of the best-defined pathways to show in which way monensin and rapamycin trigger cell death, but they may also use other death pathways in neuroblastoma cancer cells. Rapamycin is known to cause cell death by activating autophagy in human neuroblastoma cells [26]. Rapamycin can induce cell death using both apoptosis and autophagy. Therefore, rapamycin may reduce tumor volume more than monensin by using the autophagy cell death pathway.

The PI3K/AKT/mammalian target of rapamycin (mTOR) signaling pathway is one of the most potent signaling pathways that are abnormally activated in various human cancers. Recent evidence suggests that pathological activation of AKT also occurs frequently in neuroblastoma and is associated with poor prognosis. Therefore, therapeutic targeting of PI3K/AKT/mTOR may offer a promising approach for the design of molecularly targeted therapies in neuroblastoma [27]. Opel et al. examined whether activation of the PI3K pathway occurs in neuroblastoma and analyzed 116 primary neuroblastoma samples using immunohistochemistry. They reported that AKT phosphorylation, indicating activation of the PI3K/AKT/mTOR pathway, occurred in approximately two-thirds of the samples [28]. They also showed that AKT phosphorylation is associated with lower survival time [28].

Similarly, Johnsen et al. demonstrated phosphorylated AKT and mTOR expression in the cytoplasm in 30 neuroblastoma tissue samples at various stages [21]. Świeszewska et al. examined activation of the PI3K pathway in 39 samples from patients with high-risk neuroblastoma and showed that expression of PI3Kp110 and PI3Kp85 could be detected in the cytoplasm of 54% of cases, and phosphorylated AKT was present in the cytoplasm in almost all cases [29]. Our study supported that phosphorylated mTOR, PI3K, and AKT expressions were intensely expressed in the cytoplasm of the control group in the xenograft neuroblastoma model.

The PI3K/AKT/mTOR pathway regulates critical normal cellular functions such as cellular growth, proliferation, survival, and motility [30]. Components of this pathway are frequently aberrantly expressed in various tumors, making them an attractive target for anticancer therapy [30]. The pathway induces tumor growth in two ways: 1) mTOR is a vital regulator of the initiation step of autophagy, and its activation inhibits autophagy; 2) activated AKT inhibits caspase 3 and 9 by phosphorylating Ser196, ultimately inhibiting apoptosis [31,32]. Li et al. showed that the PI3K/AKT/mTOR pathway and caspase and Bcl2 signaling pathways were closely related in the DU-145 xenograft model, and treatment with AAP-H reduced p-AKT, p-PI3K, and p-mTOR protein expressions [31]. Zhang et al. showed that 20(S)-Protopanaxadiol triggered apoptosis by inhibiting the PI3K/AKT/mTOR signaling pathway in MCF-7 breast cancer cells [33]. Our study showed that the expression intensity of p-AKT, p-PI3K, and p-mTOR decreased in SH-SY5Y tumor tissues obtained from nude mice both in monotherapy groups (monensin alone, rapamycin alone) and in combination therapy groups. In addition, p-AKT, p-PI3K, and p-mTOR expression intensity were found to be lower in the combination group compared to the monotherapy groups. Our data suggest that the PI3K/AKT/mTOR signaling pathway is one of the potential mechanisms by which the combination treatment of monensin and rapamycin in vivo induces SH-SY5Y cell apoptosis.

## 4. Materials and Methods

### 4.1. Animals

Twenty-eight male nude CD1 mice, 4–6 weeks old, weighing approximately 30 g, were included in the study. The animals were cared for in laminar flow cabinets at 25 °C, on a 12 h light/dark cycle, and ad libitum access to sterile water and standard pellet food was provided. The body weights of the mice were recorded every three days. In addition, their general appearance and health status were regularly checked and recorded daily.

### 4.2. Chemicals

Monensin sodium salt, Na^+^ ionophore (Abcam, cat. no: ab120499, Boston, MA, USA), and rapamycin (MedChemEkspress, cat. no: HY-10219, Newark, NJ, USA) were dissolved in ethanol.

### 4.3. Establishment of a Xenograft Neuroblastoma Cancer Model

The SH-SY5Y (ATCC^®^-: CRL-2266™) cell line was used to establish a xenographic neuroblastoma cancer model in nude CD1 mice. The neuroblastoma model was created according to protocols previously described in the literature [9,34,35]. Briefly, SH-SY5Y cells grown, passaged, and harvested in culture medium were injected subcutaneously into the lower left abdominal quadrant of all mice in matrigel [10 µL matrigel + 90 µL PBS] and at a concentration of 10^7^ cells/mouse. SH-SY5Y injection was performed just once, and tumor formation was monitored in the mice. Tumor volume was determined using measures of the greatest length and greatest width of the tumors, obtained after measuring tumor dimensions with a caliper (tumor volume = length × width^2^ × 0.5) (Supplementary Figure S1). Measurement of tumor sizes was performed every three days. Four weeks after SH-SY5Y cell injection, 16 of the 28 injected mice met this criterion. The following stages of the research were continued with these 16 mice.

### 4.4. Formation of Experimental Groups

The 16 mice with xenograft neuroblastoma cancer model were randomly divided into four groups, namely control group, monensin group, rapamycin group, and monensin + rapamycin group.

Mice in the control group (*n* = 4) were administered 150 µL ethanol/PBS (10/100 ratio) ip (intraperitoneal) every 48 h for three weeks. Mice in the monensin group (*n* = 4) were given 10 mg/kg monensin (in 10/100 ethanol/PBS) every 48 h for three weeks, ip, and mice in the rapamycin group (*n* = 4) were given 5 mg/kg (in 10/100 ethanol/PBS) rapamycin for the same frequency and same period of time, ip. In the monensin + rapamycin group (*n* = 4), 10 mg/kg monensin and 5 mg/kg rapamycin (10/100 ethanol/PBS) were administered every 48 h for three weeks, ip.

### 4.5. Histological Evaluation of Xenograft Neuroblastoma Tumor Tissues

The mice who completed their 3-week treatment were sacrificed, and the excised tumor tissues were fixed with 4% paraformaldehyde. Tissues were embedded in paraffin after routine tissue follow-up procedures. Sections that were 5 µm thick were obtained from paraffin blocks. Tumor tissue sections obtained from all groups were stained with hematoxylin and eosin to confirm tumor development and compare the structure of tumor tissue in different experimental groups.

### 4.6. Determination of Apoptosis by TUNEL Method in Xenograft Neuroblastoma Tumor Tissues

TUNEL method was used to determine the apoptosis rate in tumor tissues. At this stage, In Situ Cell Death Detection Kit, POD (Roche, cat. no: 11684817910, Mannheim, Germany) was used, and the procedure was performed according to the protocol recommended by the manufacturer. Briefly, sections washed with PBS (phosphate-buffered saline) buffer were incubated with proteinase K solution (20 µg/mL) (Sigma-Aldrich, cat. no: 2308, St. Luis, MO, USA) for 30 min at room temperature. Then, the sections were washed with PBS and incubated with TdT (Terminal Deoxynucleotidyl Transferase) enzyme for 1 h at 37 °C. Sections washed with PBS were incubated with POD solution at 37 °C for 30 min. After washing with PBS, sections stained with DAB (3,3′-Diaminobenzidine) chromogen for 10 min were washed with PBS and counterstained with hematoxylin. In order to determine the apoptotic index (AI), four randomly selected areas in the tumor section of each animal were photographed under a light microscope at 40× magnification, and the percentage of apoptotic cells was calculated by counting TUNEL positive cells and negative cells. All these micrographs were analyzed with Image-J software.

### 4.7. Evaluation of Cleaved-Caspase 3 and p-PI3K/p-AKT/p-mTOR Protein Expression by Immunohistochemistry in Xenograft Neuroblastoma Tumor Tissues

Expression of PI3K/AKT/mTOR signaling pathway members (p-AKT, p-PI3K, and p-mTOR) and cleaved-caspase 3 in tumor tissue from xenograft mice was examined by immunohistochemistry assay. Sections were cleared with xylol and passed through different alcohol concentrations. After washing with PBS, antigen retrieval was performed with sodium citrate at 95 °C for 20 min. Subsequently, 3% H_2_O_2_/methanol was applied to block the endogenous peroxidase activity in the tissues. After washing with PBS, sections were blocked with blocking serum (Thermo Scientific, Waltham, MA, USA) and were incubated with cleaved-caspase 3 (1:2000, cat. no. BT-AP01889, BTLab, Shanghai, China), p-mTOR (1:2000, cat. no. BT- PHS00176, BTLab, Shanghai, China), p-PI3K (1:2000, cat. no. BT-PHS00765, BTLab, Shanghai, China) and p-AKT (1:2000, cat. no. BT-PHS00006, BTLab, Shanghai, China) at +4 °C for two nights. After incubation, they was washed with PBS, and a specific biotinylated goat anti-polyvalent secondary antibody was applied to the primary antibody. After washing with PBS, they were labeled with the streptavidin peroxidase enzyme complex. Proteins were made visible with the DAB chromogen. To make the nuclei visible, they were counterstained with hematoxylin, washed in tap water until the dye ran clear, and covered with entellan through increasing series of alcohols and xylol.

Micrographs were taken using a light microscope at 40× magnification. All these micrographs were analyzed with Image-J 2x.Ink software by scanning 4 nonoverlapping fields in each tissue and expressing the positive areas as a percentage of the total area. Cleaved caspase-3 positive cell numbers and the percentage of cells expressing p-AKT, p-PI3K, and p-mTOR in each tissue were calculated with the Image-J software program. Statistical significance between groups was determined using SPSS 22 software program and analyzed with TUKEY or Tamhane tests in one way ANOVA follow-up.

### 4.8. Evaluation of Cleaved-Caspase 3 and p-PI3K/p-AKT/p-mTOR Protein Expression by Immunofluorescence in Xenograft Neuroblastoma Tumor Tissues

In order to support the immunohistochemistry evaluation, the PI3K/AKT/mTOR signaling pathway in xenograft tumor tissue was examined by immunofluorescence method. Sections were cleared with xylol and passed through different alcohol concentrations. Sections were cleared with xylol and passed through different alcohol concentrations. After washing with PBS, antigen retrieval was performed with sodium citrate at 95 °C for 20 min. Subsequently, 3% H_2_O_2_/methanol was applied to block the endogenous peroxidase activity in the tissues. After washing with PBS, sections were blocked with blocking serum (Thermo Scientific, USA) and were incubated with p-mTOR (1:200, cat. no. BT-PHS00176, BTLab, Shanghai, China), p-PI3K (1:200, cat. no. BT-PHS00765, BTLab, Shanghai, China), and p-AKT (1:200, cat. no. BT-PHS00006, BTLab, Shanghai, China), for three nights at +4 °C. After incubation, they were washed with PBS and then treated with Alexa fluor 555-goat anti-rabbit (1:200, cat. no. FNSA-0095), washed with PBS. The samples were covered with an antifade mounting medium (Enzo, Farmingdale, NY, USA) and left to dry overnight. The examination of the sections marked with the immunofluorescence method was carried out on the images taken on the computer screen with a digital camera (Olympus DP71 CCD color camera, 1.5 million pixels) using the Olympus BX-FLA Reflected Light Fluorescence Attachment adapted Olympus BX50 microscope and X40 objective.

### 4.9. Statistical Analysis

Data were calculated based on the mean and standard error values of at least three independent samples. A comparison of statistical significance between groups was made using the Tukey or Tamhane methods in a one-way ANOVA follow-up. SPSS23 statistical analysis program was used, and *p* < 0.05 was considered significant.

## 5. Conclusions

This study showed that the combination of monensin and rapamycin in the xenograft neuroblastoma model significantly reduced the tumor volume, decreased the number of tumor cells, and increased the areas of fibrous connective tissue compared to the control group. In addition, the combination of monensin and rapamycin was shown to trigger apoptosis significantly and was confirmed by cleaved-caspase 3 expression. In addition, it was determined that the combination of monensin and rapamycin showed its antitumor activity by inhibiting the PI3K/AKT/mTOR signaling pathway. Our preclinical results may be a precursor to develop new therapeutic approaches that can be used in the treatment of neuroblastoma.

## Data Availability

The data supporting this study’s findings are available on request from the corresponding author.

## Supplementary Materials

The following supporting information can be downloaded at: https://www.mdpi.com/article/10.3390/antibiotics12060995/s1. Figure S1. Antitumor effects of monensin and rapamycin combination in xenograft neuroblastoma model.
