# Supplementary material for: Effects of Monensin and Rapamycin Combination Therapy on Tumor Growth and Apoptosis in a Xenograft Mouse Model of Neuroblastoma
Source: Antibiotics (Basel). 2023 Jun 1;12(6):995. doi: 10.3390/antibiotics12060995 (PMC10295421; doi:10.3390/antibiotics12060995)
Supplement: Supplementary file 1 [file antibiotics-12-00995-s001.zip › antibiotics-2395683-supplementary.pdf]

SUPPLEMENTARY MATERIAL

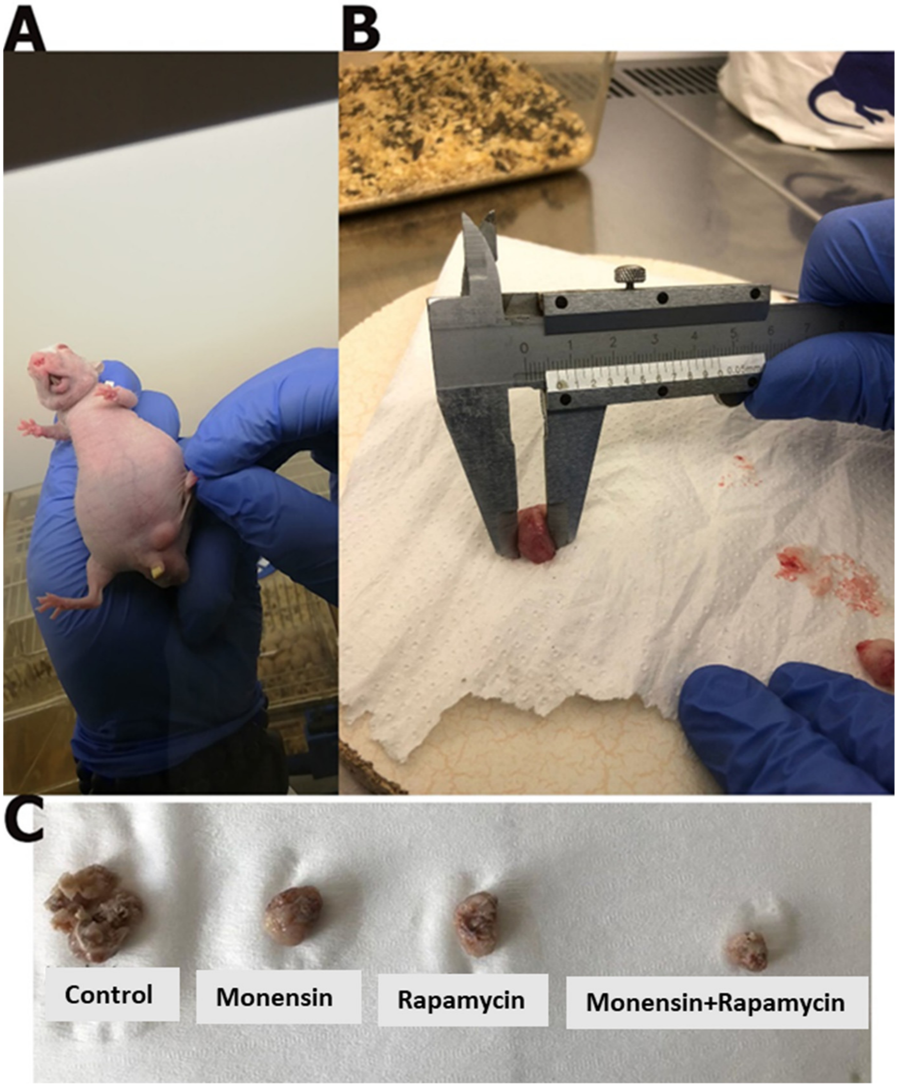

Supplementary Figure S1. Antitumor effects of monensin and rapamycin combination in xenograft neuroblastoma model. A) Xenograft neuroblastoma cancer model formation. B) Volume measurement of tumors. C) Morphological images of tumors in control, monensin, rapamycin and combination groups.
